# Establishing a business case for setting up early detection services for preventing psychosis

**DOI:** 10.1192/bjb.2022.7

**Published:** 2023-06

**Authors:** Flavia Napoletano, Olivier Andlauer, Silvia Murguia-Asensio, Savithasri V. Eranti, Elvan Akyuz, Andrés Estradé, Jonathan Buhagiar, Christine David, Paolo Fusar-Poli, Susham Gupta

**Affiliations:** 1 East London NHS Foundation Trust, London, UK; 2 Queen Mary University of London, London, UK; 3 North East London NHS Foundation Trust, London, UK; 4 King's College London, UK; 5 Catholic University, Montevideo, Uruguay; 6 South London and Maudsley NHS Foundation Trust, London, UK; 7 University of Pavia, Italy

**Keywords:** Suicide, crisis services, stigma and discrimination, service users, psychiatry and law

## Abstract

Under standard care, psychotic disorders can have limited response to treatments, high rates of chronicity and disability, negative impacts on families, and wider social and economic costs. In an effort to improve early detection and care of individuals developing a psychotic illness, early intervention in psychosis services and early detection services have been set up in various countries since the 1980s. In April 2016, NHS England implemented a new ‘access and waiting times’ standard for early intervention in psychosis to extend the prevention of psychosis across England. Unfortunately, early intervention and early detection services are still not uniformly distributed in the UK, leaving gaps in service provision. The aim of this paper is to provide a business case model that can guide clinicians and services looking to set up or expand early detection services in their area. The paper also focuses on some existing models of care within the Pan-London Network for Psychosis Prevention teams.

Psychotic disorders are associated with high levels of clinical and social morbidity, and were ranked 15th among the leading causes of disability worldwide in 2016.^1^ In England in 2011, the estimated number of new cases of psychosis ranges from 15.7 to 69.4 per 100 000 population aged 16–64 years, with an average of 24.2 per 100 000 population.^2^

Psychotic disorders usually have their onset at ages 14–35 years (median age: 25 years),^3^ being infrequent before age 14.^4^ Psychotic disorders can have a relapsing course and become chronic if not adequately managed early in their course, leading to poor interpersonal and family relationships, social exclusion, severe educational and occupational impairment, lost productivity, unemployment, various physical comorbidities, premature mortality and high rates of suicide.^5,6^ Failure to intervene early often has significant personal costs, as individuals have reduced capacity to reach their social, emotional and vocational potential.^7^ Psychotic disorders also come at a significant price to society in terms of poor economic and social participation, high clinical and social care expenditure as well as carers’ burden. The global economic burden of schizophrenia is estimated to range from 0.02 to 1.65% of gross domestic product.^8^ In England alone, the estimated annual cost of schizophrenia is £11.8 billion to society and £7.2 billion to the public sector (2010–2011 prices), when all costs are considered.^9^

The management of the early phase of psychosis can be critical and influence long-term clinical, social and functional outcomes.^10^ Early intervention in psychosis (EIP) services provide specialised and comprehensive support at the time of the first psychotic episode.^11^ Clinical evidence from meta-analyses confirms the superiority of multicomponent EIP interventions compared with standard community care on various outcomes, including all-cause treatment discontinuation, psychiatric hospital admission, involvement in school or work, total symptom severity, positive symptom severity and negative symptom severity.^12^ Specialised EIP services can also reduce service costs by about 35% for adults^13^ and 27% for children and adolescents^14^ compared with generic services, mainly by lowering readmission rates despite higher costs of the EIP teams. In the UK, the success and cost-effectiveness of first-episode services led to their recommendation in National Institute for Health and Care Excellence (NICE) clinical guidelines for both adults^15^ and young patients.^16^ However, EIP services have limited impact on reducing the duration of untreated psychosis,^17^ which is a core determinant of long-term outcomes. Furthermore, EIP services can only deliver secondary prevention and are therefore less likely to benefit vulnerable people who are at risk of developing a first episode of psychosis but are not yet unwell and therefore need a primary indicated prevention (Table 1).^18^

**Table 1:** World Health Organization's classification of preventive approaches for mental disorders

Public health classification of prevention	Gordon's classification of prevention, modified by the US Institute of Medicine
*Primary prevention* seeks to prevent the onset (incidence) of a disorder or illness	
	*Universal prevention* is defined as those interventions that are targeted at the general public or a whole population group that has not been identified on the basis of increased risk
	*Selective prevention* targets individuals or subgroups of the population whose risk of developing a mental disorder is significantly higher than average, as evidenced by biological, psychological or social risk factors
	*Indicated prevention* targets high-risk people who are identified as having minimal but detectable signs or symptoms foreshadowing mental disorder, or biological markers indicating predisposition for mental disorders, but who do not meet diagnostic criteria for disorder at that time
*Secondary prevention* seeks to lower the rate of established cases of the disorder or illness in the population (prevalence) through early detection and treatment of diagnosable diseases	
*Tertiary prevention* includes interventions that reduce disability, enhance rehabilitation and prevent relapses and recurrences of the illness	

© 2021 World Psychiatric Association. Reproduced with permission of the WPA from Fusar-Poli P, Correll C, Arango C, Berk M, Patel V, Ioannidis J. Preventive psychiatry: a blueprint for improving the mental health of young people. *World Psychiatry* 2021; **20**: 200–21.

## Moving towards indicated prevention: early detection services

Attempts have been made for a long time to identify a clinical phase preceding the onset of psychosis for prevention of a potential first episode.^19^ Real-world indicated prevention was made possible with the introduction of the clinical high-risk state for psychosis (CHR-P) paradigm more than two decades ago in Melbourne, Australia.^20^ Specialised early detection services – as they are known in the UK – have since been established to identify and provide preventive (indicated) interventions and specialised assessment to individuals meeting CHR-P criteria.^21^ A recent survey identified 47 CHR-P services worldwide offering care to 22 248 individuals in Western Europe (51.1%), North America (17.0%), East Asia (17.0%), Australia (6.4%), South America (6.4%) and Africa (2.1%).^22^ The CHR-P criteria identify three potential groups of individuals with an increased risk of developing psychosis: (a) those with attenuated psychotic symptoms; (b) those with brief limited intermittent psychotic symptoms (BLIPS); and (c) those at genetic risk or with trait vulnerability in addition to a drop in functioning for at least 1 month within the previous year.^23^ CHR-P individuals are help-seeking^24^ and services are typically delivered to young people between 14 and 35 years old.

CHR-P status is established via semi-structured clinical interviews such as the Comprehensive Assessment of At-Risk Mental States (CAARMS), which was developed by the Melbourne group,^25^ and the Structured Interview for Prodromal Symptoms (SIPS) and the associated Scale of Prodromal Symptoms (SOPS), more commonly used in the USA and other international centres.^26,27^ The overall accuracy of CHR-P assessment instruments to predict psychosis onset (area under the curve AUC = 0.90) is comparable to other tests used in preventive medicine.^28^ However, the predictive value of these tools is low in non-clinical samples who have not undergone risk enrichment (see below).^29^

Young people meeting CHR-P criteria for genetic and/or clinical risk factors for psychosis^26,27,30,31^ have approximately a 50-fold overall increase in risk of developing a psychotic disorder, compared with the general population.^32^ Overall risk of transition has been estimated to be 19% at 12 months, 19% at 24 months, 25% at 36 months and 28% at ≥48 months.^33^ Among CHR-P subgroups, individuals with BLIPS constitute the group with the highest risk of transition to psychosis: 22% at 12 months, 39% at 24 months, 38% at 36 months and 38% at ≥48 months. This is followed by the those with attenuated psychotic symptoms (16, 19, 21 and 24% respectively at the four time points). Individuals at genetic risk or with trait vulnerability do not appear to have an enhanced risk compared with the general population.^34^

It is worth noting, however, that 45% of non-transitioning CHR-P individuals will continue to present functional impairment at 6 year follow-up.^35^ Efforts continue to refine assessments to increase the predictive power in identifying transition to psychosis. Potential future strategies include the use of sequential assessments based on clinical and biomarker data, and more effective recruitment to enhance pretest psychosis risk (so-called risk enrichment) in clinical samples.^36^

## Evidence base for early detection services

Interventions that delay or prevent transition to psychosis are considered valuable at both the economic and the individual level,^37,38^ and this is particularly evident in younger people who are at clinically high risk of developing psychosis and facing a potentially lifelong chronic illness. Hence, there has been a drive for expansion of early detection services, as reflected in their inclusion in clinical guidelines at a national^15,16,39,40^ and international^41^ level, and in diagnostic manuals.^42^ In the UK, NICE guidelines^15,16^ and the National Health Service (NHS) Access and Waiting Time (AWT) standards^43^ recommend the provision of specialised assessment, individual and family psychosocial interventions, and support for comorbid conditions to young individuals meeting CHR-P criteria. Community outreach and education should also be conducted to ensure that young people at risk of psychosis are picked up and referred to early detection services.^43^

Various interventions, such as cognitive–behavioural therapy (CBT), antidepressants, low-dose antipsychotic medication and omega-3 fatty acids, have been researched.^30^ The International Early Psychosis Association (IEPA) recommends regular monitoring of mental state, targeted interventions for specific difficulties (such as anxiety, depression, substance misuse), support with interpersonal, vocational and family stress, help in developing coping abilities for subthreshold symptoms, and individual and/or family psychoeducation.^39^ NICE recommends that these interventions should ideally be provided in low-stigmatising environments (such as at home, or in primary care or youth-based settings) and in a flexible manner that promotes access. Despite these attempts, meta-analytic evidence (including a recent Cochrane review) does not support the superiority of any individual intervention over another or over control condition (needs-based interventions) to prevent psychosis transition in at-risk individuals.^44,45^

Benefits of early detection services extend beyond prevention of transition to psychosis. Individuals who do not make the transition usually have mental health problems^46,47^ and they may also benefit from early identification and intervention. For example, there are several clinical outcomes beyond the development of psychosis that are not currently targeted by available interventions.^48^ At-risk individuals frequently present with comorbid mental health conditions that are addressed by early detection services as part of their standard care package.^10,42^ Early detection services also provide vocational support,^49^ reduce family burden and stress,^10^ and improve trust and engagement.^50^

Other potential indirect benefits, such as a positive initial experience of contact with psychiatric services, are likely to have longer-term protective effects through more voluntary and less coercive engagement with individuals at risk of psychosis (and their families). Among at-risk individuals transitioning to psychosis, early detection services may be effective in reducing the duration of untreated psychosis and the need for hospital admission following psychosis onset.^51^ This can help to reduce trauma associated with formal detention and involvement of the police and criminal justice system during crisis. If successful, this may be particularly important for engaging people from Black, Asian and minority ethnic backgrounds, given their often negative experience of psychiatric services, and poor clinical and social outcomes.

From an economic perspective, early detection services can be potentially cost-effective, given the huge personal and long-term social costs associated with psychotic disorders, especially in young people.^52^ Real-world evidence supports the long-term (24-month follow-up) cost savings for early detection services.^53^

## Setting up an early detection service: moving towards a more preventive model of care

Although the evidence for EIP services is clearly established, and they are now an integral part of the psychiatric care landscape, specialised CHR-P teams are still in their relative infancy, with most early detection services having been created since 2000.^21^ There is substantial heterogeneity in how these services operate in the UK and around the world, in the absence of clear operational guidance.^21,54^

The Pan-London Network for Psychosis Prevention (PNP)^55^ was established in 2017 in an effort to promote awareness, knowledge and good practice among existing early detection services. The PNP is working towards developing guidance and operational policies from existing best evidence and clinical experience.

In the following sections, we present the business case for an early detection service that can be used by services in England and other parts of the UK. This is based on current experiences of existing models of care locally.

In England, the government's Five Year Forward View recommended early intervention for mental illness, with the shift towards preventive healthcare intervention being the primary driver of change.^56^ Recent initiatives, such as sustainability and transformation partnerships, primary care networks, the integrated neighbourhood model, and the children and young people plan, can all be incorporated at local level for developing a comprehensive community-based model. The importance of improving youth mental health is advocated in the current mental health strategy,^57^ which embraces a shift in focus of services towards promotion of mental health, prevention of mental illness, early identification and intervention across the life-course.

Furthermore, in the current NHS Mental Health Implementation Plan^58^ it is stated that all areas of England will need to ensure they are commissioning EIP services in line with NHS England guidance, including providing a service that covers an age range of 14 to 65 years and has a provision for individuals meeting the CHR-P criteria. Improvements in concordance with NICE guidelines are also expected in line with this trajectory.^58^

### Components of an early detection service

The success of the CHR-P paradigm is determined by the concurrent integration of three core components: focusing on efficient detection of at-risk cases, accurate prognosis and effective preventive treatment.^10,51,55,59–61^ The main components of such a service are as follows.

#### Outreach component

To identify at-risk young people at an early stage, help-seeking needs to be promoted by working together with local communities and maintaining liaison with statutory and non-statutory stakeholders and partners. Barriers to accessing services, such as stigma or taboos, need to be addressed, for example by improving awareness and providing psychoeducation for both front-line workers and the wider community. The outreach component draws on a public health model with the aim of increasing the likelihood of access and help-seeking. This involves working with all potential stakeholders in the community to help to identify young people who may be at risk of developing psychosis and other mental health problems. This sits between primary and secondary prevention.

#### Specialist assessment component

Specialist assessment sits at the interface between primary care and specialist services. High-quality assessment in non-stigmatising environments is able to identify those at high risk of developing psychosis or to transfer those already experiencing psychosis to the EIP service. Those not at risk can be signposted to other support through statutory or non-statutory/third-sector organisations.

#### Specialist mental health intervention component

This involves the delivery of a wide range of psychosocial interventions, as well as medical care that attends to the needs of those at risk; this aims to provide ongoing monitoring and a biopsychosocial model of care to reduce the risks of transition to psychosis. As mentioned above, an early detection service can also facilitate prompt transfer of care to the EIP service for those who transition to psychosis, reducing the duration of untreated psychosis.

### Important considerations

An early detection service should aim to reach the entire population within a catchment area and make it possible for early identification and access to care for people meeting CHR-P criteria. For greater effectiveness such strategies should be able to positively target groups who are often over-represented in EIP services, such as people with a history of adverse experiences/trauma, migrant groups, people using drugs and alcohol, and other potentially vulnerable groups. This requires evaluation of the local population to identify high-risk groups, along with the mapping of locations that can facilitate outreach to these groups in a positive and non-stigmatising way. Although most of the literature is on those who seek help, more assertive outreach work may have benefits in high-risk marginalised groups through more socially focused strategies.

It is also vital to develop strong working relationships with primary care (general practitioner practices) and statutory and non-statutory services such as schools, youth centres, prisons, supported accommodation, spiritual care services, community centres and community leaders. Hence, the physical location of such services is very important in improving access. Ideally, they should be based in the community and away from mental health bases and clinics.

From the outset, it would be important to identify the position of the early detection service within a local healthcare structure, given its unique position transcending public health, primary care and specialist mental health services.

### Funding

Funding availability depends on the sociopolitical climate nationally and locally. With the introduction of the National Service Framework in the UK in 1999,^62^ there was a new direction in the NHS. The next important step was in 2000, when the government set out a 3-year plan identifying three health priority areas, one of which is mental health. An extra £300 million annual investment was released by 2003–2004 for development of specific services. There was an aim to set up 50 EIP services by 2004.^63^ Further to this there was a road map for development of EIP services nationally in all boroughs, and funding followed this goal. Any such national framework provides a great opportunity and ensures local development of services following the pre-defined road map in all areas in the country.

The early intervention movement at this time had consumer support from patient and carer groups, with champions canvassing for the movement. Further to this there was a strong evidence base for the clinical and cost-effectiveness of early intervention over the following 10 years.

In 2014, the Five Year Forward View^56^ brought in new funding to achieve various targets, which included a target for referral to treatment within 2 weeks and provision of NICE-based interventions for all patients with a first episode of psychosis. There was also an increased demand for expansion of EIP services as increased rates of psychosis were shown in urban and immigrant populations.^64,65^ The evidence base for this helped further develop websites such as PsyMaptic (www.psymaptic.org), which gives an incidence calculator for each borough in England.^66^ Funding streams were directed based on needs of local boroughs. This historical perspective in the UK illustrates the importance of national priorities, research evidence, and patient and carer involvement in determining funding priorities.

Currently, the NHS Long Term Plan^67^ focuses on young people aged 14–25 years and the Mental Health Transformation Programme integrates primary and secondary care mental health services. There is an opportunity here for youth-focused prevention work within these frameworks if local stakeholders engage with it. If key targets are set out by the government for early detection and prevention of psychosis, it is likely that funding will be available and the development of such services locally will be possible.

Against this background, service arrangements at local level within clinical commissioning and sustainability and transformation partnership structures need discussion, as early detection services straddle public (preventive), primary and secondary services. Local stakeholders need to be involved in discussions on allocation of resources. In some areas, third-sector organisation involvement might be prominent and this can be harnessed in the development of early detection services. Some of the funding can be non-recurrent and could be used to pilot early detection services.

### Models of early detection services

There are currently several different models of early detection services.

Stand-alone teams in the community, separate from secondary mental health services, are possibly the most desirable model to remove barriers to access to care.^21^ Locating such bases in non-clinical settings is likely to reduce stigma and to encourage help-seeking. Stand-alone services have strong links with primary care settings where the initial assessment and some care can be provided.

A second option is to have early detection services that are integrated within EIP services. Sharing a physical location with established psychiatric clinics can be a barrier for reasons mentioned previously.

A third option for early detection services is to adopt a ‘hub and spoke’ model of care in conjunction with partner organisations. In hub and spoke models, early detection workers are based in generic mental health services and refer patients needing more intensive treatment to the central ‘hub’. Empirical evidence and results from the Pan London Network for Psychosis Prevention survey^55^ clearly show that stand-alone teams are more established and successful than integrated and hub and spoke teams. As teams in the hub and spoke model share their resources with other mental health services, staff time can be easily taken up by the needs of the most severely ill patients rather than those meeting CHR-P criteria.

### Age range

Based on the highest likelihood of emergence of early-onset psychotic disorder, the most frequent target population in the UK ranges between the ages of 12 (lower end) and 35 (upper end) years.^21^ Clinical and medico-legal aspects of providing care for under-18s would need to be considered. Joint working provisions with child and adolescent mental health services (CAMHS) would require local service level arrangements and reviews.

Current variations in models of care include, at the lower end:

- services for over-18s
- services for over-18s with the option of joint working with CAMHS patients and young people coming to the end of their time with CAMHS
- under-18s who require input from the CAMHS team, especially regarding prescribing of medication and potential admission to hospital.

At the upper end, the current range is 25–35 years.

The age range of the target population will have a bearing on case-loads and overall costs.

### Referral sources and case-loads

A mapping of potential sources of referrals needs to be undertaken to identify local stakeholders and to make a projection of the potential case-load. Appropriate utilisation of internet-based platforms can increase access via self-referrals, while also promoting psychoeducation and offering other resources.

An effective early detection service should have a projected case-load that is reflective of the local population and the rate of psychosis. It should be able to identify those at highest risk of transition to psychosis (high positive predictive value). Even those not transitioning to psychosis should also benefit from interventions offered.

### Identification of partners and stakeholders

An early detection service should aim to collaborate with existing services to identify those at risk, maximise outreach potential, reduce costs and avoid duplication of resources.

Potential stakeholders include:

- primary care and primary care psychiatric liaison services, which are possibly the largest source of referrals
- CAMHS: a comprehensive local strategy is needed for the under-18 subpopulation accommodating variations in local strategies
- educational institutions: schools and colleges cover a substantial target population, and good co-working relationships help with identification and with access, while supporting patients to remain in education
- emergency and crisis pathways such as accident and emergency (A&E) and psychiatric liaison services
- substance misuse services
- the police and criminal justice system and probation services
- social services and local authorities, because of their role in supporting vulnerable families, children of people with severe mental disorders and other vulnerable people
- youth, sports and recreational facilities and activity coordinators
- online and digital platforms accessed by the local population
- churches and other community centres: engagement of community leaders can improve access to specific ethnic and cultural subgroups
- youth peer support programmes and local initiatives.

### Duration of care under early detection services

Worldwide, duration of care offered by CHR-P services is most commonly around or less than 2 years.^21^ This falls below the current clinical evidence, which indicates the need for monitoring of clinical outcomes for at least 3 years to maximise chances of identifying transition to psychosis of those at risk and avoiding missing long-term outcomes.^32^ A duration of 2 years is insufficient to map all long-term needs of this population. We think that it would be beneficial having a service which is able to look after at-risk individuals for at least 3 years, with a flexible discharge plan depending on clinical needs.

### Team composition and workforce

The NHS clinical reference group for EIP services devised a workforce calculator based on case-loads and service delivery requirements. A similar approach could be used to work out the composition of professionals within early detection services. A multidisciplinary team is needed to provide specialist assessments, bespoke psychosocial interventions and medical support. Provisions should exist for maintaining links with potential referrers and other services, for providing psychoeducation and training to these partner organisations and stakeholder services, and for keeping media platforms up to date.

#### Expert assessors

Specialist training is required to carry out the initial clinical assessment using validated tools that can be administered by mental health and social care workers, with psychology support and supervision. Qualified trainers can also offer training and supervision to appropriate professionals from other services.

#### Keyworkers

Patients are usually allocated a keyworker from the service. Keyworkers can be from different specialties and should be able to provide some standardised psychosocial and practical support in the community. This includes psychoeducation, physical and mental health promotion, smoking cessation, harm reduction work on substance use, education and employment support, and liaising with educational institutions and employers.

#### Outreach and liaison workers

Outreach work can be delivered by trained staff who can provide psychoeducation and liaison with various stakeholders to facilitate appropriate referrals.

#### Specialist psychological interventions

Psychological interventions are considered^37^ the mainstay of early detection services and aim at improving clinical and functional outcomes. Psychologists with specific training in this area should provide a wide range of psychological interventions, such as CBT (including for psychosis), family work and trauma-informed interventions. Non-psychologists with training can provide some interventions under psychology supervision.

#### Medical care

Medical support is needed for psychiatric evaluation, providing diagnosis and pharmacological treatment if indicated. The role of medical staff includes risk assessment and crisis management, as well as clinical management of those transitioning to psychosis. Patients under the age of 18 would require specialist CAMHS support.

#### Management

A service manager or a team leader is expected to oversee the management of the service along with ensuring that regular and appropriate clinical supervision is offered to team members.

#### Peer and support workers

These can be an invaluable resource for outreach work and providing peer-to-peer support.

### Assessing outcomes and monitoring service performance

It may be a challenge to set specific clinical outcome measures for new early detection services owing to the wide-ranging and complex clinical presentation of an at-risk population and local variations. Performance targets may have to be locally benchmarked based on an initial assessment of local needs and the size of target population. Benchmarking and auditing of existing clinical outcomes can help incremental improvement in service delivery through local learning.

Individual progress can be monitored using patient-reported experience measures (PREMs) and patient-reported outcome measures (PROMs) such as DIALOG+ (which is a simple intervention to assess life and treatment satisfaction of patients and address concerns) and the Health of the Nation Outcome Scales (HoNOS). Other measures could include those based on social functioning.

## Conclusions

As the UK government aims to promote a more preventive healthcare system, early detection services should be an important component of the mental health structure. There is gathering evidence to support the setting up of such services but, in the absence of clear funding streams as well as wide variations in existing models, there is a need for a more standardised approach. Until this is achieved, it may be helpful to learn from existing models and to support the development of new services, which involves securing funding from commissioners. We hope that this article and the business case template in the Appendix below can be used as a resource in such negotiations.

## Data Availability

Data availability is not applicable to this article as no new data were created or analysed in this study.

## About the authors

**Flavia Napoletano**, Consultant Psychiatrist, Newham Early Intervention Service (NEIS), East London NHS Foundation Trust, London, UK. **Olivier Andlauer**, Consultant Psychiatrist, HEADS UP, East London NHS Foundation Trust, London; and Centre for Psychiatry, Wolfson Institute of Preventive Medicine, Barts and the London School of Medicine and Dentistry, Queen Mary University of London, UK. **Silvia Murguia-Asensio**, Consultant Psychiatrist, Tower Hamlets Early Detection Service (THEDS), East London NHS Foundation Trust, UK. **Savithasri V. Eranti**, Consultant Psychiatrist, Newham Early Intervention Service (NEIS), East London NHS Foundation Trust, UK. **Elvan Akyuz**, Consultant Psychiatrist, Redbridge Early Intervention in Psychosis Service, North East London NHS Foundation Trust, UK. **Andrés Estradé**, Research Assistant, Early Psychosis: Interventions and Clinical-detection (EPIC) Lab, Department of Psychosis Studies, Institute of Psychiatry, Psychology & Neuroscience, King's College London, UK; and Department of Clinical and Health Psychology, Catholic University, Montevideo, Uruguay. **Jonathan Buhagiar**, Clinical Psychologist, HEADS UP, East London NHS Foundation Trust, UK. **Christine David**, Operational Lead, Tower Hamlets Early Detection Service (THEDS), East London NHS Foundation Trust, UK. **Paolo Fusar-Poli**, Consultant Psychiatrist, Early Psychosis: Interventions and Clinical-detection (EPIC) Lab, Department of Psychosis Studies, Institute of Psychiatry, Psychology & Neuroscience, King's College London; and OASIS Outreach and Support Service, South London and Maudsley NHS Foundation Trust, London, UK; and Department of Brain and Behavioural Sciences, University of Pavia, Italy; and National Institute for Health Research, Maudsley Biomedical Research Centre, South London and Maudsley NHS Foundation Trust, London, UK. **Susham Gupta**, Consultant Psychiatrist, HEADS UP, East London NHS Foundation Trust, UK.

## Supplementary material

For supplementary material accompanying this paper visit https://doi.org/10.1192/bjb.2022.7. click here to view supplementary material

## Appendix

We suggest the following business case template for setting up early detection in psychosis services.

**Table:**

Executive summary
Introduction
**Strategic background** Evidence base for early detection
**Strategic context** Add national and local objectives
**The case for change** Clinical effectiveness (better clinical care through improved prognosis and reducing chronicity) Cost-effectiveness (reducing burden on healthcare systems and better socioeconomic participation of affected individuals) Better patient experience (e.g. less stigma, easier accessibility, less restrictive care) Reduced costs for services (reduce duplication and improving co-working with existing services and third-sector stakeholders) Population and public health benefits, including health promotion Corporate social responsibility (involving local community and longer-term potential for recruiting local people) Incorporating the ‘green agenda’ (e.g. through better IT systems and offering a range of modes of consultations)
**Investment objectives** Local SMART (specific, measurable, achievable, realistic and timed) indicators
**Current service provision** Assessment of existing mental healthcare landscape to identity potential gaps in services Transition from CAMHS to adult services
**Proposal for service** Describe proposed local service structure
**Drivers for the new service and sustainability plan** Describe local strategy and involvement of commissioners
**Deliverable outcomes** Describe locally agreed outcome measures
**Strategic risks** Potential low uptake (identify possible risks and how to mitigate) Possible duplication of existing services Any potential harm or other unintended consequences on existing services Staffing resources, training, recruitment barriers
**Funding and affordability** State clearly the intended source of funding including preferred options Elicit support from commissioners and outcomes from initial negotiations
**Funding risks** Clear plan on funding options and identification of potential financial risks, e.g. risks associated with unclear or non-recurrent funding streams. Also ensure that funding is not diverted from other necessary services including early intervention services. Constraints and dependencies: risk of multiple source of funding with fixed contracts
**Preferred option** State the preferred option and why
**Other options** Option 1: Do nothing or do minimum – non-compliant with national guidance and Five Year Forward View strategies Option 2: Public health/presentation – lack of specialist training and psychosocial and medical support Option 3: Primary care: lack of specialist training and psychosocial and medical support Option 3: Secondary mental health services – stigma, labelling, access only when really unwell, worse outcomes
**PREMS/PROMS** Local measures National measures, e.g. DIALOG+, the Health of the Nation Outcome Scales (HoNOS)
**Management arrangements, accountability and governance** Include clear clinical and management structure (can be complicated with varying funding structures) and accountability
**Facilities and buildings**
**Workforce** Composition Training
**Timeline** Timetable for delivery of project
**Organisational change and change management** How to monitor aims, and increase benefits and reduce or minimise risks and ‘dis-benefits’ through audits, service evaluations and impact assessments
**Evaluation of project**
**References**
**Appendices**
