# Supplementary material for: Establishing a business case for setting up early detection services for preventing psychosis
Source: BJPsych Bull. 2023 Jun;47(3):156–65. doi: 10.1192/bjb.2022.7 (PMC10214435; doi:10.1192/bjb.2022.7)
Supplement: Supplementary file 1 [file S2056469422000079sup001.docx]

**Table 2.** **A business case template for Early Detection in Psychosis services**

| EXECUTIVE SUMMARY:  1.1 Introductions: |
| --- |
| STRATEGIC BACKGROUND:   - Evidence base for ED   **Strategic Context:**   - Add National and Local objectives   **The Case for Change:**   - Clinical Effectiveness (better clinical through improved prognosis and reducing chronicity) - Cost Effectiveness (reducing burden on healthcare systems and better social-economic participation of affected individuals) - Better Patient experience (E.g. less stigma, easier accessibility, less restrictive care) - Reduced costs for services (reduce duplication and improving co-working with existing services and third-sector stakeholders) - Population and Public health benefits including health promotion - Corporate social responsibility (Involving local community and longer-term potential for recruiting local people) - incorporating the Green Agenda (e.g. through better IT systems and offering a range of modes of consultations   Investment Objectives:   - Local SMART (Specific, Measurable, Achievable, Realistic and Timed) indicators |
| CURRENT SERVICE PROVISION:   - Assessment of existing mental health care landscape to identity potential gaps in services - CAMHS transition |
| PROPOSAL FOR SERVICE:  Describe proposed local service structure |
| DRIVERS FOR THE NEW SERVICE AND SUSTAINABILITY PLAN:  Describe local strategy and involvement of commissioners |
| DELIVERABLE OUTCOMES:  - Describe locally agreed outcome measures |
| STRATEGIC RISKS:  Potential low uptake (identify possible risks and how to mitigate)  Possible duplication with other existing services  Any potential harm or other unintended consequences on existing services  Staffing resources, training, recruiting barriers |
| FUNDING AND AFFORDABILITY:  State clearly the intended source of funding including preferred options.  Elicit support from commissioners and outcomes from initial negotiations |
| FUNDING Risks:  Clear plan around funding options and identification of potential financial risks – e.g. risks associated with unclear or non-recurrent funding streams. Also ensure funding is not diverted from other necessary services including Early Intervention Services.  Constraints and Dependencies:  Risk of multiple source of funding with fixed contracts |
| PREFERRED OPTION:  OTHER OPTIONS:   - Option 1: Do nothing or do minimum – non-compliant with national guidance and five years forward view strategies - Option 2: Public Health/Presentation: lack of specialist training and psychosocial and medical support - Option 3: Primary Care: lack of specialist training and psychosocial and medical support - Option 3: Secondary MH services: stigma, labelling, access only when really unwell, worse outcomes |
| PREMS/PROMS:  Local measures  National measures e.g. DIALOG+, the Health of the Nation Outcome Scales (HoNOS) amd other validated tools. |
| MANAGEMENT ARRANGEMENTS, ACCOUNTABILITY AND GOVERNANCE:  Include Clear Clinical and Management structure (can be complicated with varying funding structures) and accountability |
| FACILITIES AND BUILDINGS: |
| WORKFORCE:   - COMPOSITION - TRAINING |
| TIME-LINE:   - Time-table for delivery of project |
| ORGANISATIONAL CHANGE AND CHANGE MANAGEMENT:  How to monitor aims, and increase benefits and reduce and minimise risks and “dis-benefits” through audits, service evaluations and impact assessments |
| EVALUATION OF PROJECT: |
| References |
| Appendices |
